# HER2 testing results, practices, and preferences among pathologists and oncologists in the US community setting: a mixed-methods study

**DOI:** 10.1007/s10549-025-07832-1

**Published:** 2025-11-21

**Authors:** Simon M. Collin, Clara Lam, Simone T. Sredni, Zakiya M. Haji-Noor, Miriam J. Haviland, Lisa Okazaki, Edward Espinal-Dominguez, John D. Cochran, Angel F. Valladares, Marija Tesic-Schnell

**Affiliations:** 1 Oncology Outcomes Research, AstraZeneca Pharmaceuticals Ltd, Cambridge, UK; 2 US Medical Affairs, AstraZeneca Pharmaceuticals LP, Gaithersburg, MD USA; 3 IQVIA Real World Solutions, New York, NY USA; 4 Cancer Biomarker Development, Oncology R&D, AstraZeneca Pharmaceuticals LP, Gaithersburg, MD, USA; 5 Oncology Center of Excellence - Therapeutic Science and Strategy Unit (TSSU), IQVIA, Frankfurt, Germany

**Keywords:** Breast cancer, Human epidermal growth factor receptor 2 (HER2), Immunohistochemistry (IHC), Pathology, HER2-directed therapy

## Abstract

**Purpose:**

To quantify the proportion of HER2-negative metastatic breast cancers with low or ultralow levels of HER2 expression and identify facilitators and barriers to HER2 testing and reporting in US community settings.

**Methods:**

Analysis of electronic medical record data from a retrospective cohort of patients diagnosed with HER2-negative breast cancer from 2018 to 2023 within the Guardian Research Network, classifying HER2 status by immunohistochemistry (IHC) score. Analysis of responses to surveys of community-based pathologists and oncologists, supplemented by qualitative analysis of one-to-one interview transcripts.

**Results:**

The retrospective study identified 13,824 patients diagnosed with HER2-negative breast cancer from seven healthcare organizations, with 13,100 patients included in the final cohort. Patients were classified as HER2 IHC 0 (32%), 1 + (35%), 2 + (18%), and 3 + (1%); 15% of patients did not have a documented IHC score. Surveys and interviews with 63 community-based pathologists and oncologists found that most pathologists (93%) reported discrete IHC scoring on pathology reports, but 16% had difficulty assigning scores between IHC 0 and IHC 1 + . Barriers included inadequate standards, increased interpretation time, and workflow disruptions. Digital pathology was used by 39% of pathologists, with improved accuracy, higher efficiency, and reduced subjectivity stated as advantages, and high costs and lack of practice standards as barriers to adoption.

**Conclusion:**

While innovative testing tools were viewed favorably by pathologists and oncologists, cost and need for training were barriers to adoption. Improving documentation practices, standardizing protocols, and adopting tools such as digital pathology could enhance the accuracy and consistency of HER2 testing.

**Supplementary Information:**

The online version contains supplementary material available at 10.1007/s10549-025-07832-1.

## Introduction

**Fig. 1:**
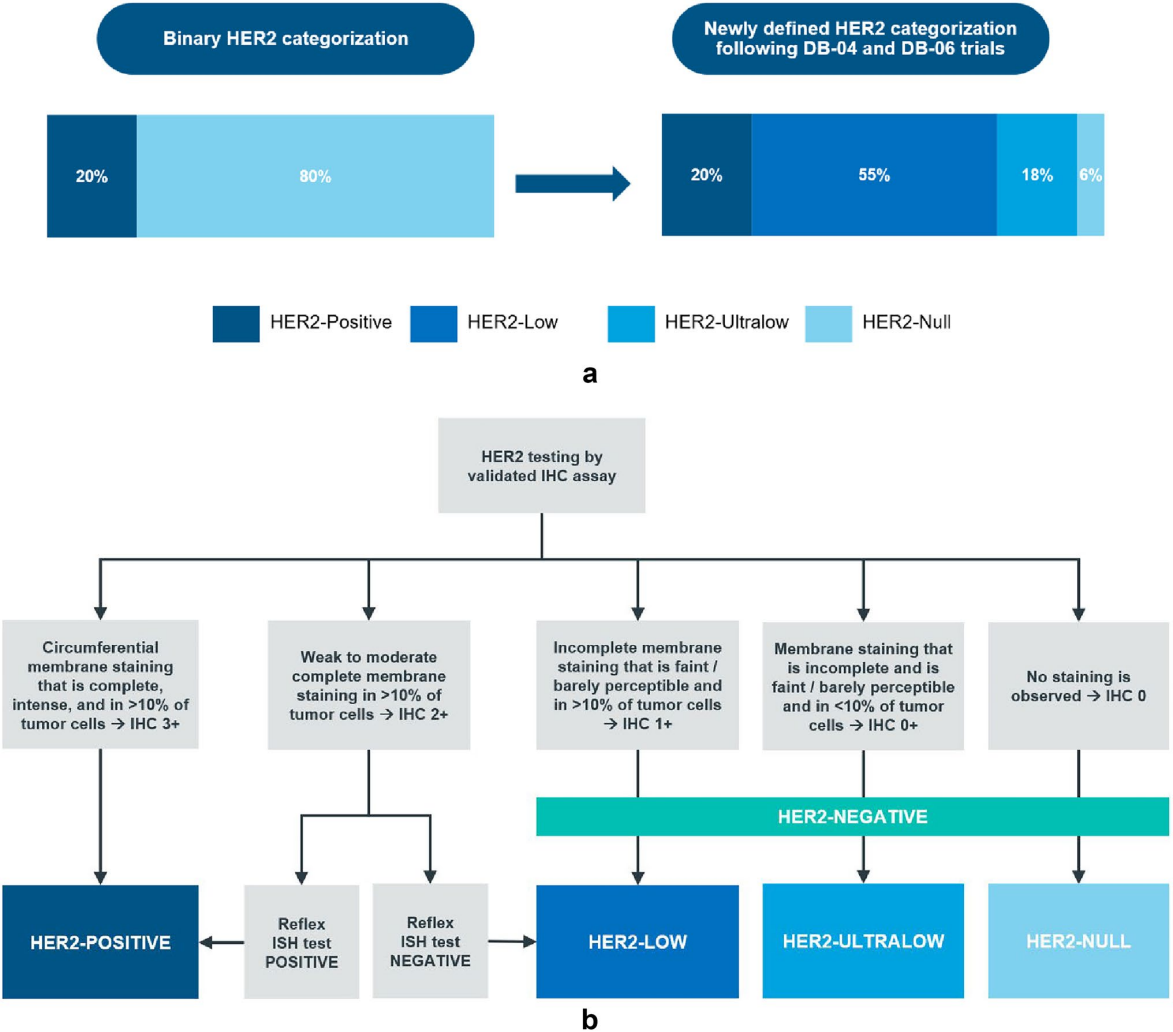
**a** Evolution of HER2 IHC categorization to include HER2-low and HER2-ultralow categories ^a^*DB-04* DESTINY-Breast04, *DB-06* DESTINY-Breast06, *HER2* human epidermal growth factor receptor 2, *IHC* immunohistochemistry. ^a^Data sources: Refs. [9–12]. **b** HER2 evaluation by IHC and ISH testing ^a^*HER2* human epidermal growth factor receptor 2, *IHC* immunohistochemistry, *ISH* in situ hybridization. ^a^Data source: Reference [3]

Human epidermal growth factor receptor 2 (HER2) is a protein that is overexpressed because of *HER2* (*ERBB2*) gene amplification and plays a crucial role in the development and progression of breast cancer [1]. Until recently, HER2 status was reported as either positive or negative (Fig. 1a), with only HER2-positive patients being eligible for HER2-directed therapies [2]. HER2-directed therapies are now known to be effective in treating tumors that exhibit low or even very low expression of HER2 that were previously classified as HER2-negative (Fig. 1a, b) [3, 4].

The DESTINY-Breast04 (DB-04) trial demonstrated that trastuzumab deruxtecan (T-DXd, Enhertu) vs physician’s choice of chemotherapy improved median progression-free survival (PFS) by 4.8 months and median overall survival by 6.6 months in patients with HER2-low (Fig. 1b) unresectable and/or metastatic breast cancer who had received one or two prior lines of chemotherapy [4].

Accurate and timely determination of a patient’s HER2 status is therefore essential to identifying appropriate treatment [5]. In 2023, the American Society of Clinical Oncology (ASCO) and College of American Pathologists (CAP) published updated HER2 testing guidelines [2], confirming (per previous guidelines) that pathology reports should include the immunohistochemistry (IHC) score and a footnote to identify patients with HER2 IHC 1 + or 2 + who may be eligible for therapies that target lower levels of HER2 expression. The 2023 ASCO-CAP guidelines list best practices to distinguish IHC 1 + from IHC 0 [2].

DB-04 was followed by DESTINY-Breast06 (DB-06), which demonstrated improved PFS for T-DXd vs physician’s choice of chemotherapy (13.2 vs 8.1 months) in patients with HER2-low status who had received one or more lines of endocrine therapy and similar improvement in an exploratory group of patients with HER2-ultralow status (Fig. 1b) [3]. Based on the DB-04 and DB-06 trial findings, Enhertu was approved in 2022 as the first HER2-directed therapy for patients with HER2-low metastatic breast cancer who have received a prior chemotherapy in the metastatic setting or developed disease recurrence during or within 6 months of completing adjuvant chemotherapy, and in 2025 as the first HER2-directed therapy for patients with HER2-low or HER2-ultralow metastatic breast cancer following disease progression after one or more endocrine therapies. Accordingly, the most recent ASCO-CAP template updates on 19 March 2025 provided updated guidance for pathologists to report HER2-ultralow, i.e., presence vs absence of staining in IHC 0 (https://documents.cap.org/documents/New-Cancer-Protocols-March-2025/Breast.Bmk_1.6.0.0.REL.CAPCP.pdf).

The pace of change in HER2-directed therapeutic options and related precision medicine testing requirements by global regulatory agencies creates a challenge for pathologists who determine HER2 scoring and oncologists who use information from pathologists to determine treatment options for patients. In particular, while community-based oncology practices enable patients to benefit from more personalized, convenient, and cost-effective care [6], these settings may have fewer resources, including for training in new guidelines and technological advancements, fewer opportunities for medical professionals to specialize, and more diverse patient populations than are encountered in academic centers [4, 7, 8]. These challenges may result in practice gaps such as inconsistent documentation and a lack of standardized reporting of HER2 IHC and in situ hybridization (ISH) status.

Our study applied quantitative and qualitative methods to understand the extent to which practice gaps existed (post-DB-04 but pre-DB-06) and to explore the reasons for these gaps in a network of community healthcare organizations (HCOs) across the US. We conducted a retrospective cohort study to quantify, by breast cancer stage (early, locally advanced, metastatic), the proportion of cases identified in electronic medical records (EMRs) as ‘HER2-negative’ who had low or ultralow levels of HER2 expression. In parallel, we used online surveys and individual interviews to describe current practices (post-DB-04, pre-DB-06) and elucidate oncologists’ and pathologists’ perceptions around HER2 testing, and to identify facilitators and barriers to (ASCO-CAP 2023) guideline-supported HER2 IHC testing, reporting, and treatment in the community setting.

## Methods

### HER2 IHC test results

The retrospective cohort study used an electronic medical record (EMR) database within the Guardian Research Network (GRN), a research network of community hospitals across the US. Full methodological details are described in Online Resource 1. In brief, data were abstracted from medical records of patients at four healthcare organizations (HCOs) within GRN comprising 43 cancer centers and 85 hospitals across 15 states. Patients were included if they were ≥ 18 years old, diagnosed with breast cancer, had ≥ 1 clinical evaluation related to their cancer from 1 January 2018 to 15 November 2023, and had documentation of HER2-negative breast cancer status. A natural language processing (NLP) algorithm was applied to structured and unstructured EMR fields to identify HER2-negative status. A patient was considered to have HER2-negative breast cancer if, at any time during the study period, they had documentation of the following: 1) HER2 IHC 0 or IHC 1 + or IHC 2 + with ISH-negative; 2) HER2 status noted as HER2-negative or HER2-low; or 3) HER2 status associated with negative qualitative values for IHC or ISH results, such as “Not positive”, “Negative”, “HER2 0”, “HER2 1 + ”, and “Not expressed”. Patients with conflicting HER2 test results per the NLP algorithm that could not be resolved by manual investigation of medical records were not classified as HER2-negative and therefore were not eligible for inclusion in the study cohort. HER2 IHC scores were abstracted from unstructured pathology reports using an NLP algorithm. Patients with a documented IHC score were classified into subgroups (IHC 0, IHC 1 + , IHC 2 + , and IHC 3 +) based on the highest IHC score documented during the study period. Demographic and clinical characteristics were abstracted from structured EMR fields.

### Healthcare professional surveys and interview data

#### Study participants

The survey and interview analysis consisted of responses from US community-based pathologists and oncologists recruited through GRN and IQVIA’s Healthcare Professional (HCP) panel. Full methodological details are described in Online Resource 2. In brief, HCPs who self-reported involvement in biomarker testing and/or treatment of breast cancer patients in a community setting in the US were eligible for inclusion. Pathologists were classified as general or specializing in breast cancer or surgical pathology [13–15]. Oncologists were either medical oncologists or breast oncologists [16]. Two separate structured web-based surveys were developed (one for oncologists, one for pathologists) with input from subject matter experts, supported by preliminary results from the retrospective cohort EMR analysis. Survey responses were collected between February 2024 and May 2024 and respondents were invited to participate in semi-structured interviews. Interviews were conducted from March 2024 to May 2024, using interview guides tailored to pathologists or oncologists annotated with each HCP’s survey response to facilitate discussion. Interviews were analyzed using a thematic analysis approach to highlight key themes and patterns in the data.

## Results

### HER2 IHC test results

**Fig. 2:**
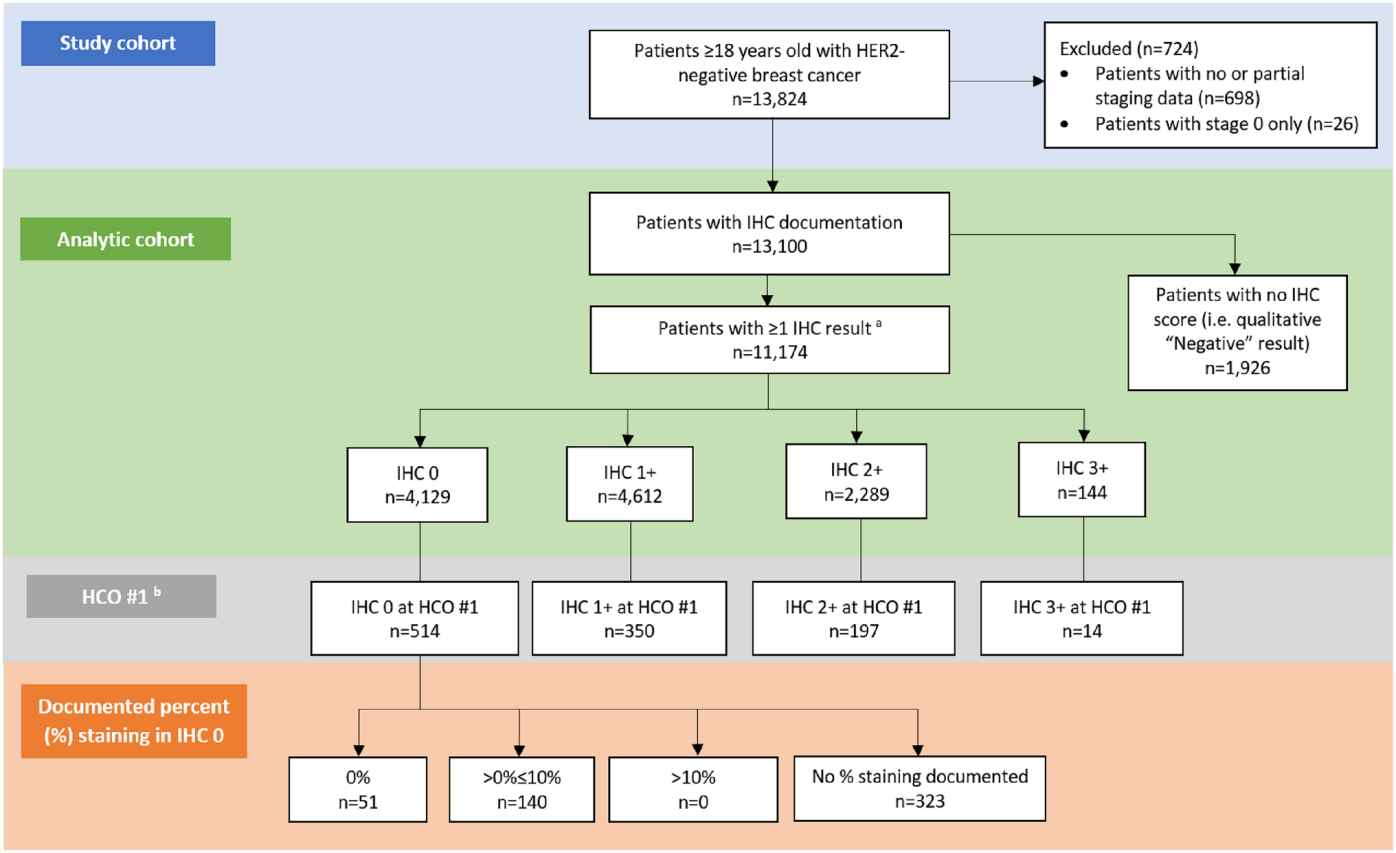
HER2-negative breast cancer patient selection flowchart based on EMR data from patients treated at GRN HCOs. *EMR* electronic medical record, *GRN* Guardian Research Network, *HCO* healthcare organization, *HER2* human epidermal growth factor receptor 2, *IHC* immunohistochemistry. ^a^For patients with ≥ 1 IHC score, the highest score was used. ^b^Four patients at HCO#1 had no IHC score documented

The study cohort comprised 13,824 HER2-negative breast cancer patients from seven HCOs within GRN (n = 298 patients were excluded from the study cohort during manual evaluation because they had conflicting results which the NLP algorithm could not resolve). After excluding 724 patients with incomplete staging data (n = 698) or carcinoma-in-situ / pre-invasive disease (stage 0) breast cancer (n = 26), 13,100 patients were eligible for inclusion in the analytic cohort (Fig. 2); n = 265 patients with more than one HER2 result during the study period were retained in the analytic cohort. Among the 13,100 patients included in the analytic cohort, the highest documented IHC score during the study period was 0 for 4,129 (31.5%) patients, 1 + for 4,612 (35.2%) patients, 2 + for 2,289 (17.5%) patients, and 3 + for 144 (1.1%) patients. The remaining 1,926 (14.7%) patients did not have a numeric IHC score documented in pathology records, although they did have documentation of being HER2-negative in their medical records (e.g., a qualitative value of “HER2-negative”) (Table 1). ISH results for patients whose highest score was IHC 2 + are provided in Online Resource 3; all patients with IHC 2 + and ISH-positive as highest score were confirmed to have documentation of HER2-negative status during the study period. The results of a sensitivity analysis wherein patients were classified into subgroups based on the lowest IHC score documented during the study period are provided in Online Resource 4.

**Table 1:** HER2 IHC score by breast cancer stage in HER2-negative cohort based on highest IHC score documented during the study period^a^

Among patients at all HCOs after exclusion criteria are applied	All HER2-negative n = 13,100	Early-stage HER2-negative n = 10,746	Locally advanced HER2-negative n = 355	Metastatic HER2-negative n = 1,999
IHC 0	4,129 (31.5%)	3,369 (31.4%)	118 (33.2%)	642 (32.1%)
IHC 1 +	4,612 (35.2%)	3,845 (35.8%)	98 (27.6%)	669 (33.5%)
IHC 2 + ^b^	2,289 (17.5%)	1,786 (16.6%)	73 (20.6%)	430 (21.5%)
IHC 3 + ^c^	144 (1.1%)	105 (1.0%)	4 (1.1%)	35 (1.8%)
No IHC score documented ^d^	1,926 (14.7%)	1,641 (15.3%)	62 (17.5%)	223 (11.2%)
Among patients at HCO #1	**All HER2-negative** **n = 1,079**	**Early-stage HER2-negative** **n = 951**	**Locally advanced HER2-negative** **n = 48**	**Metastatic HER2-negative** **n = 80**
All IHC 0	514 (47.6%)	452 (47.5%)	27 (56.2%)	35 (43.8%)
IHC 0 absent membrane staining (HER2-null; 0% staining)	51 (9.9%)	43 (9.5%)	3 (11.1%)	5 (14.3%)
IHC 0 with membrane staining (HER2-ultralow; 0% < staining ≤ 10%)	140 (27.2%)	130 (28.8%)	2 (7.4%)	8 (22.9%)
Percent staining not documented	323 (62.8%)	279 (61.7%)	22 (81.5%)	22 (62.9%)
IHC 1 +	350 (32.4%)	313 (32.9%)	11 (22.9%)	26 (32.5%)
IHC 2 + ^b^	197 (18.3%)	174 (18.3%)	9 (18.8%)	14 (17.5%)
IHC 3 + ^c^	14 (1.3%)	10 (1.1%)	0 (0.0%)	4 (5.0%)
No IHC score documented ^d^	4 (0.4%)	2 (0.2%)	1 (2.1%)	1 (1.3%)

*AJCC* American Joint Committee on Cancer, *HCO* healthcare organization, *HER2* human epidermal growth factor receptor 2, *ICD-10* International Statistical Classification of Diseases and Related Health Problems, 10th Revision, *IHC* immunohistochemistry

^a^Breast cancer stage within + / − 90 days of IHC test result. Early-stage breast cancer: AJCC stages IA, IB, IIA, IIB, or IIIA or corresponding TNM staging; locally advanced breast cancer: stages IIIB, IIIC, or corresponding TNM staging; metastatic breast cancer: ICD-10 codes C77, C78, C79; AJCC stage IV; TNM M1; or NLP-derived “Stage IV” or “M1” disease

^b^ISH results for patients whose highest score was IHC 2 + are provided in Online Resource 3; all patients with IHC 2 + and ISH-positive as highest score were confirmed to have documentation of HER2-negative status during the study period

^c^All patients with IHC 3 + documented as highest score were confirmed to have documentation of HER2-negative status during the study period

^d^All patients without a documented IHC score had qualitative documentation of HER2-negative status in their medical record (e.g., HER2 status = ‘Negative’)

Percent staining of invasive tumor cells among IHC 0 patients was documented only at HCO #1. Among the 514 patients at HCO #1 classified as IHC 0, 191 (37.2%) had percent staining documented while 323 (62.8%) did not (Table 1). Of those with documented percent staining, 73.3% were HER2-ultralow and 26.7% were IHC 0 absent membrane staining (Fig. 2).

### HCP surveys and interviews

#### HCP characteristics

Surveys were conducted among 63 US community-based pathologists and oncologists (Table 2, Online Resource 5), of whom 27 were interviewed. Although various factors may have influenced survey/interview responses, such as familiarity with recent findings from clinical trials and ASCO-CAP guideline updates, oncologists and pathologists agreed that oncologists’ preferences and requests (which included the level of details in pathology reports, requests for retesting, and alignment on interpretation) were the strongest drivers of changes in HER2 testing and treating practices. For example, a pathologist from a large community health system noted that oncologists request “*That the pathologists be customer service oriented and do tests in specific ways*.”

**Table 2:** Pathologists’ and oncologists’ practitioner characteristics

Domain	n (%) – Surveys	n (%) – Interviews
*Pathologists*	(N = 31)	(N = 14)
**Focus of practice**		
Breast pathologists	23 (74.2)	8 (57.1)
General pathologists	6 (19.4)	4 (28.6)
Surgical pathologists	2 (6.5)	2 (14.3)
Laboratory directors	N/A	7 (50.0)
**Practice setting **^*a*^		
Community	N/A	10 (71.4)
Satellite of AMC		5 (35.7)
Independent practice		1 (7.1)
Cancer center		3 (21.4)
IDN		2 (14.3)
**Familiarity with DB-04 results **^*b*^		
High familiarity	N/A	7 (50.0)
Limited familiarity		3 (21.4)
Unknown		4 (28.6)
*Oncologists*	**(N = 32)**	**(N = 13)**
**Focus of practice**		
Breast oncologists	3 (9.4)	1 (7.7)
Medical oncologists	29 (90.6)	11 (84.6)
Not reported	0 (0)	1 (7.7)
**Practice setting**^*a*^		
Community	N/A	8 (61.5)
Satellite of AMC		2 (15.4)
Independent practice		3 (23.1)
**Have you practiced post-residency at an AMC?**		
Yes	26 (81.3)	8 (61.5)
No	6 (18.8)	4 (30.8)
Unknown	0 (0)	1 (7.7)
**Breast cancer case volume**		
Low (< 10%)	N/A	0 (0)
Moderate (10–40%)		7 (53.8)
High (> 40%)		5 (38.5)
Not reported		1 (7.7)

*AMC* academic medical center, *ASCO* American Society of Clinical Oncology, *DB-04* DESTINY-Breast04, *IDN* Integrated Delivery Network

^a^Practice settings are not mutually exclusive

^b^Survey responses were collected between February and May 2024; interviews were conducted from March to May 2024. DB-04 topline results were announced on 21 February 2022; complete results were presented at the ASCO Annual Meeting in June 2022 and published in The New England Journal of Medicine at the same time[4]

### Pathologists’ practices and preferences

#### Analytic practices

Pathologists reported varied analytic practices, including location of lab testing, assays used, and reporting practices (Table 3). Among surveyed pathologists, 16.1% (n = 5) did not distinguish between IHC 0 and IHC 1 + and 29.0% (n = 9) did not report percent staining. A pathologist who does not report percent staining explained: “*Percent staining is not quantified because it doesn’t add any new information and is more tedious, requiring an increase in interpretation time as a barrier*.” Among pathologists who do report percent staining, there were challenges in: “*Evaluating immunostain[ing]*” because “*in one area that I’m evaluating it could look like more than 10% if I count one high power field … [but] in the different field it could look like less than 10%.*” When asked about testing and reporting, one pathologist who divides their time between a community hospital, cancer center, and academic medical center (AMC) compared institutions: “*The main driving factor for the differences [in institutions’ testing practices] is purely economic. NCI [National Cancer Institute] cancer centers are very well funded … [whereas] in community hospitals, the [patients’] insurance is not very good,*

**Table 3:** Pathologist analytic practices

Domain	n (%) (N = 31)
**Location of lab testing**	
Outsourced	4 (12.9)
In-house	27 (87.1)
Hybrid	0 (0.0)
**Assay used** ^a^	
DAKO	8 (25.8)
Ventana	16 (51.6)
DAKO + Ventana	6 (19.4)
Not reported	1 (3.2)
**2023 ASCO-CAP guidelines**	
High familiarity	19 (61.3)
Limited familiarity / somewhat familiar	11 (35.5)
Not reported	1 (3.2)
**HER2 IHC scoring**	
Distinguishes between IHC 0 and IHC 1 +	25 (80.6)
Does not distinguish between IHC 0 and IHC 1 +	5 (16.1)
Not reported	1 (3.2)
**HER2 percent staining in IHC 0**	
Reports percent staining	22 (71.0)
Does not report percent staining	9 (29.0)
Not reported	0

*ASCO-CAP* American Society of Clinical Oncology–College of American Pathologists, *HER2* human epidermal growth factor receptor 2, *IHC* immunohistochemistry

^a^Ventana’ refers to VENTANA Anti-HER2/neu (4B5) Rabbit Monoclonal Primary Antibody assay; ‘DAKO’ refers to HercepTest™ mAb pharmDx (Dako Omnis)

#### Reporting, retesting, collaboration, and digital/emerging technologies

Surveyed pathologists reported several barriers to IHC and ISH testing and reporting (Table 4): background or faint staining by 9.7% (n = 3); heterogeneity or staining variability by 22.6% (n = 7); and standardized interpretation by 16.1% (n = 5). These barriers were noted by interviewed pathologists (n = 14): “*The difference between 0 and 1* + *can be really difficult and subjective.*” Reasons for retesting were: random / quality control (16.1%, n = 5); known or suspected discrepancy (35.5%, n = 11); when IHC = 0 (12.9%, n = 4); and when IHC is equivocal (51.6%, n = 16). One pathologist noted that retesting is done in their lab: “*If the tumor is now being graded at a higher grade than the initial [result],*” while another answered: “*If the patient progress[es] [even with] treatment, then we do re-biopsy … [to see] if there is any transformation of the receptors.*”

**Table 4:** Pathologists’ barriers and facilitators to reporting practices

Domain	n (%) – Surveys (N = 31)^a^
*Drivers and barriers to reporting*	
**Barriers to reporting discrete IHC scores**	
Background/faint staining	3 (9.7)
Standardized interpretation	5 (16.1)
Heterogeneity / staining variability	7 (22.6)
Increase in interpretation time / workflow disruption	1 (3.2)
**Reason for retesting (multi-select question)**	
Random / quality control	5 (16.1)
Known or suspected discrepancy	11 (35.5)
When IHC = 0	4 (12.9)
When IHC is equivocal	16 (51.6)
**Collaboration (multi-select question)**	
Tumor board	27 (87.1)
With other pathologists	6 (19.4)
With oncologists	4 (12.9)
With pathologists and oncologists	13 (41.9)
Limited/none	7 (22.6)
*Emerging/digital technologies*	
**Use**	
Currently using	12 (38.7)
Does not use	18 (58.1)

*IHC* immunohistochemistry

^a^Some survey respondents did not answer all questions

Pathologists reported collaboration with other HCPs as a means of reconciliation of discrepant testing results as well as an avenue for remaining updated with professional organizations’ guidelines and recommendations and research findings. Pathologists collaborated with other HCPs primarily through tumor boards (87.1%, n = 27, of surveyed pathologists (a tumor board is a multidisciplinary team that meets to review patient cases and discuss treatment plans). Pathologists shared during interviews that tumor boards at their institutions were either a mix of general oncology cases, breast-specific tumor boards, or they designated specific sessions to breast cancer cases; frequency of tumor boards ranged from biweekly, to weekly, to monthly. One oncologist whose institution utilizes a regular tumor board said: “*If there’s confusion [regarding testing results], we can always reach back; that’s why we have a tumor board*.”

During interviews, seven (50.0%) pathologists shared that testing practices did not change following the 2023 guideline update as their practices were already aligned to the recommendations. When asked about challenges to adopting changes to reporting, including discrete IHC scores and percent staining, interviewees indicated that inclusion of this detail was contingent on requests by oncologists. One pathologist commented on the need for ‘buy-in’ from oncologists: “*The oncologists would have to strongly get on board with it and we don’t have very many requests for that here*.”

Digital pathology had been adopted by 38.7% (n = 12) of surveyed pathologists. Of interviewed pathologists, 50% (7/14) reported the potential for these emerging technologies to close gaps and remove barriers to HER2 IHC testing and reporting as a reason for their adoption. Emerging technologies reported to be in use included next-generation sequencing (50.0%, n = 7), digital imaging/scanning tools (28.6%, n = 4), or other emerging or digital tools (28.6%, n = 4). Pathologists cited improved accuracy, increased efficiency, and reduced subjectivity as perceived advantages to the use of digital pathology tools. However, pathologists also commented on considerations for adopting new technologies including pricing, billing, technical requirements, lab staff, additional equipment, quality assurance/controls, and adoption practices of AMCs or cancer centers. Cited barriers to adoption included cost and absence of practice standards. Moreover, there were indications of resistance to adoption based on how the tools are implemented: “*We have tried digital analysis. It saves time, and it enhances the confidence of the pathologists, but I stopped it because the operation needed third or fourth parties other than the pathologists*” and “*digital pathology is not run by the software, and not run by pathologists, but by administrative assistance.*” Owing to the lack of established best practices for using digital pathology tools, some pathologists are hesitant to adopt them until more evidence is available.

### Oncologists’ practices and preferences

Most oncologists reported receiving discrete IHC scores, with 96.9% (31/32) of surveyed oncologists confirming this practice (Table 5). Reasons for requesting retesting included the need for more granular details to determine treatment eligibility (reported by 25.0%, n = 8, of surveyed oncologists), determining current HER2 status of metastatic cases (reported by 15.6%, n = 5), and discordant results (reported by 40.6%, n = 13).

**Table 5:** Oncologists’ surveys

Domain	N (%) – Surveys (n = 32)^a^
**Receives discrete IHC scores**	
Yes	31 (96.9)
No	1 (3.1)
Not reported	0 (0)
**Rerun requests**	
For more granular details (distinguish IHC 0/IHC 1 + or to request percent staining) / to determine treatment eligibility	8 (25.0)
For metastatic or recurrent cases	5 (15.6)
For discordant results	13 (40.6)
**2023 ASCO-CAP Guidelines**	
High familiarity / received ASCO training	17 (53.1)
Limited/no specialized training	14 (43.8)
Not reported	0 (0)
**Tumor board**	
Yes	23 (71.9)
No	8 (25.0)
Not reported	1 (3.1)

*ASCO-CAP* American Society of Clinical Oncology–College of American Pathologists, *IHC* immunohistochemistry

^a^Some survey respondents did not answer all questions

Among interviewed oncologists, 23.1% (3/13) reported receiving reports with percent staining documented, 53.8% (n = 7) without,, and interviewed oncologists emphasized the importance of IHC score compared with percent staining: “*To be honest, I don’t focus on those percentages. Usually it’s enough for us [to make treatment decisions based on] 1*+ *or 2*+.” Another oncologist echoed this sentiment, stating that percent staining reporting “*Does not make a difference in clinical practice.*”

Many interviewed oncologists indicated that they were already following the 2023 ASCO-CAP guidelines, and therefore no major changes to their treating processes were made: “[*We] did it before I heard about that (the 2023 guideline update).*” Regarding their perspective on the importance of HER2 testing guideline updates, one preferred to know: “*Whether I can use the drug or not.*” Overall, oncologists noted that they trust pathologists on their reporting and testing processes. As one interviewed oncologist put it: “*I really trust pathology … I mistrust myself in pathology because I’m not trained*.” However, approval of HER2-directed therapies was reported as the primary driver to changes in treatment recommendations, as reported by 84.6% (n = 11) of interviewed oncologists: “*The approval of HER2-targeted therapy, especially for HER2-low patients, has been a game changer. It has expanded our treatment options and allowed us to offer more personalized care.*” Another interviewed oncologist shared this opinion, saying: “*The approval of HER2-targeted therapy has provided new hope for patients who previously had limited options*.”

#### Factors affecting treatment decision-making

More than a quarter (29.0%, n = 9) of surveyed oncologists cited reimbursement of tests and treatments as major barriers to treatment decision-making: “*We deal with different kinds of insurance, like Medicare, Medicaid, private insurance, Tricare, VA, and everything, so it’s hard to convince the insurance sometimes, and you don’t want patients to have copays, which can be in thousands of dollars*.” Tissue paucity, accuracy of HER2 testing results, and HER2-low status were reported by 21.9% (n = 7) of surveyed oncologists as a barrier to treatment decision-making.

Feedback from 23.1% (n = 3) of interviewed oncologists and 14.3% (n = 2) of interviewed pathologists in this study indicated that these HCPs were supportive of the use of HER2-directed therapies for all patients with metastatic breast cancer where appropriate, regardless of HER2 expression levels.

## Discussion

Data from EMRs of US community health systems complemented by findings from surveys and interviews of practitioners highlight variability in testing practices and reporting of HER2 IHC scores. These include lack of discrete score documentation, lack of distinction in the lower range of HER2 status, and lack of consensus definitions being applied consistently across HCOs. Our findings align with previous studies that have reported challenges in HER2 testing and reporting practices [17, 18].

HCP interviews revealed challenges driving gaps in discrete IHC score documentation including issues with background/faint staining, a lack of standardized interpretation, and heterogeneity/staining variability. The variability in documentation practices suggests a lack of standardization in reporting. This gap underscores the need to continue to engage pathologists with resources and education to support improved adherence to documentation and reporting practices. This might include joint oncologist/pathologist peer-to-peer educational outreach from larger academic centers to their regional / local communities and awareness-raising campaigns. Given advancements in HER2-directed therapies in breast cancer, detailed documentation is instrumental in guiding treatment decisions and facilitating future research in these patient populations.

### Adherence to ASCO-CAP guidelines

The gap in distinguishing HER2 status at lower levels of expression may have impacted oncologists’ decision-making, because at least a quarter of surveyed oncologists mentioned that they had to request IHC retesting or rescoring to differentiate IHC 0 from IHC 1 + , as specified in guidelines at the time of the surveys and interviews. These deviations and delays could be barriers to timely access to targeted therapies. These challenges have been previously reported [19], highlighting the need for standardized protocols and training to improve the accuracy and consistency of HER2 testing.

FDA approval of HER2-directed therapies in the ‘ultralow’ group will require differentiation of HER2 IHC 0 absent membrane staining vs IHC 0 with membrane staining. The most recent CAP template updates on 19 March 2025 provide updated guidance for pathologists to report HER2-ultralow, i.e., IHC 0 with membrane staining that is incomplete and is faint / barely perceptible and in ≤ 10% of tumor cells. ASCO-CAP guidelines at the time of our study did not require the reporting of the estimated percent staining, and only one center in our study reported HER2-ultralow vs HER2-null (and for < 40% of patients). Although the key aspect is to report presence vs absence of staining, i.e., it is not critical to report estimated percent staining in test results, this significant practice change will likely present further challenges for pathologists (and oncologists).

### Potential barriers to testing

Pathologists highlighted technical challenges, workflow disruptions, lack of standardization, and increased time required for evaluation as barriers to consistent discrete IHC reporting. In distinguishing IHC 0 from IHC 1 + , staining variability and background staining and the subjective nature of IHC interpretation were noted as issues. These findings underscore the need for improved training and standardized protocols to enhance the accuracy and consistency of how HER2 status is reported, particularly in the community setting. Given the clinical relevance of HER2 IHC status on the lower end of the spectrum for patients with metastatic breast cancer, reliable and timely biomarker testing is critical for treatment access [19].

The absence of documented IHC score at some centers may have been due to delays incorporating the latest guidelines/templates from National Comprehensive Cancer Network or CAP into laboratory information systems and/or lack of integration with EMR systems. Additional data are needed on new assays currently in development to evaluate their ability to measure HER2 protein expression quantitatively and to ensure they can do so consistently and reliably once they are validated and standardized.

### Emerging tools and solutions

Our study explored HCP perceptions of emerging technologies, including digital pathology and AI algorithms, and their potential to address some of the challenges associated with HER2 testing. While digital pathology was viewed favorably by almost all of the interviewed oncologists, only half of interviewed pathologists shared this view. Pathologists interested in emerging technology and tools are under-resourced to invest in these, especially in the absence of validated precedence for the technologies’/tools’ utility in community-based institutions where resources are often limited. The perceived advantages of digital pathology, including improved accuracy and increased efficiency, suggest that digital pathology could play a significant role in enhancing HER2 testing practices. Several studies corroborate this perspective of efficiency and operational utility provided by digital pathology [20, 21]. However, cost and lack of practice standards were identified as significant barriers to widespread adoption [22, 23].

### Strengths and limitations

In utilizing EMR data from GRN, this study captured and characterised patients with HER2-negative breast cancer across several HCOs, including patients with HER2-ultralow status from one HCO. We also captured perspectives from oncologists and pathologists in community settings. HCPs and their practices were diverse in their specialties, geographic location, and overall perspectives, providing a comprehensive view of HER2 IHC testing and reporting practices in real-world clinical settings.

We used a small convenience sample of HCPs and our findings may not be generalizable to all community HCPs in the US or to academic oncology centers. HCPs self-selected to participate in surveys and interviews, which may have introduced participation bias. The design of data collection and interpretation of themes and patterns may have been influenced by the researchers’ own perspectives and preconceptions. EMR data, as is the case for real-world data, are subject to missingness and misclassification. For example, some patients were excluded from the analytic cohort because their breast cancer stage was not recorded. Although most of the data extracted via the NLP algorithm were validated manually (approximately 95%), there may be some misclassification of patients as HER2-negative and by IHC subgroup. HER2 IHC methods are not necessarily consistent with one another or used similarly between institutions (or between pathologists). For example, the primary HER2 antibodies in each assay method are different, with different affinities for their antigenic site, and antigen retrieval conditions used in different institutions are likely different, which would be expected to produce somewhat different IHC staining results. HER2 IHC scoring is subjective, and differences in HER2 IHC staining are not necessarily related consistently to low levels of HER2 protein expression. Qualitative and quantitative data were collected over different time periods and from different health systems, meaning that our methods were not ‘mixed’ sensu stricto. In particular, membrane staining data were available from a single center from which we were unable to secure an interview with a pathologist to understand the reasons for the reporting of these data. Finally, our study was conducted post-DB-04 but pre-DB-06 (which indicated benefit of HER2-directed therapies at very low levels of HER2 expression), and when ASCO-CAP 2023 guidelines were current (before the 2025 reporting template update specifying reporting of absence vs presence of staining in IHC 0) hence, our findings will not reflect the very latest practices and perceptions of HCPs with regard to HER2 testing and reporting.

### Conclusions

Our study highlights significant variability in HER2 IHC testing and reporting practices in community settings, as well as diverse perceptions and practices among pathologists and oncologists. Addressing the identified barriers to guideline-supported HER2 testing and reporting, including improving documentation practices, standardizing protocols, and leveraging emerging tools such as digital pathology and AI, could improve the consistency of HER2 testing and reporting. These improvements will be essential for optimizing treatment decisions and outcomes for breast cancer patients, a large proportion of whom receive their oncology care in the community setting.

## Supplementary Information

Below is the link to the electronic supplementary material.

Supplementary file1 (PDF 334 KB)

## Data Availability

The datasets generated during and/or analysed during the current study are not publicly available due to data license restrictions (GRN EMR data) and confidentiality (survey responses and interview transcripts) but are available from the corresponding author on reasonable request.
