# Supplementary material for: HER2 testing results, practices, and preferences among pathologists and oncologists in the US community setting: a mixed-methods study
Source: Breast Cancer Res Treat. 2025 Nov 21;215(1):2. doi: 10.1007/s10549-025-07832-1 (PMC12638377; doi:10.1007/s10549-025-07832-1)
Supplement: Supplementary file 1 — Supplementary file1 (PDF 334 KB) [file 10549_2025_7832_MOESM1_ESM.pdf]

Online resources

Identification of HER2-low and HER2-ultralow breast cancer: a mixed-methods study of HER2 testing results, practices, and preferences among pathologists and oncologists in the community setting

Breast Cancer Research & Treatment

Simon M. Collin[1*‡] · Clara Lam[2*] · Simone T. Sredni[2] · Zakiya Haji-Noor[3] · Miriam J. Haviland[3] · Lisa Okazaki[3] · Edward Espinal Dominguez[3] · John D. Cochran[3] · Angel F. Valladares[3] · Marija Tesic-Schnell[2]

[1] Oncology Outcomes Research, AstraZeneca Pharmaceuticals Ltd, Cambridge, , UK

[2] US Medical Affairs, AstraZeneca Pharmaceuticals LP, Gaithersburg, MD, USA

[3] IQVIA Real World Solutions, New York, NY, USA

* Joint first author

‡ Corresponding author simon.collin@astrazeneca.com

Online Resource 1

Methods for HER2 IHC and ISH test result data abstraction and analysis

Online Resource 2

Methods for HCP surveys and interviews

Online Resource 3

ISH result by breast cancer stage among patients in HER2-negative cohort with IHC 2+ score based on highest IHC score documented during the study period

Online Resource 4

HER2 IHC score by breast cancer stage in HER2-negative cohort based on lowest IHC score documented during the study period

Online Resource 5

Geographic distribution of surveyed HCPs across the US by HCP type

Online Resource 1

Methods for HER2 IHC and ISH test result data abstraction and analysis

Study population and patient inclusion

The retrospective cohort study used an EMR database within the Guardian Research Network (GRN), a research network of community hospitals across the US. Data for this study were abstracted from medical records of patients at seven HCOs within GRN comprising 40+ cancer centers and 90+ hospitals. Patients from GRN were included in the study cohort if they were ≥18 years old, diagnosed with breast cancer (International Statistical Classification of Diseases and Related Health Problems, 10th Revision [ICD-10] code C50), had ≥1 clinical evaluation related to their cancer from 1 January 2018 to 15 November 2023, and had documentation of HER2-negative breast cancer status.

A natural language processing (NLP) algorithm was applied to structured and unstructured EMR fields to identify HER2-negative status. A patient was considered to have HER2-negative breast cancer if, at any time during the study period, they had documentation of the following: 1) HER2 IHC 0 or IHC 1+ or IHC 2+ with ISH-negative; 2) HER2 status noted as HER2-negative or HER2-low; or 3) HER2 status associated with negative qualitative values for IHC or ISH results, such as “Not positive”, “Negative”, “HER2 0”, “HER2 1+”, and “Not expressed”. Patients with conflicting HER2 test results per the NLP algorithm that could not be resolved by manual investigation of medical records were not classified as HER2-negative and therefore were not eligible for inclusion in the study cohort. Patients were excluded from the analytic cohort if they had stage 0 breast cancer, or incomplete documentation of staging data such that their stage could not be determined in the analysis. Patients whose HER2 status changed during the study period, e.g., changed from HER2-negative to HER2-positive, and who met all other eligibility criteria were included in the analytic cohort.

Demographic and clinical data

Demographic and clinical characteristics for all patients included in the analytic cohort were abstracted from structured EMR fields, including age, sex, race and ethnicity, Charlson comorbidity index score, and progesterone and estrogen status at time of IHC score.

HER2 IHC test result data

HER2 IHC scores were abstracted from unstructured pathology reports using an NLP algorithm. Patients with a documented IHC score were classified into subgroups based only on the highest IHC score documented during the study period. These HER2 IHC subgroups were IHC 0, IHC 1+, IHC 2+, and IHC 3+. Although IHC 3+ is associated with HER2-positive status, the HER2 status of some patients included in the analytic cohort was recorded more than once, such that their highest IHC score during the study period was 3+. Similarly, abstraction of ISH scores for patients who were ‘HER2-negative’ with an IHC score of 2+ revealed some patients whose ISH result was positive, hence ‘HER2-positive’. Feasibility analyses had determined that only one HCO (referred to as HCO #1) documented percent staining of invasive tumor cells among IHC 0 patients. Patients at this HCO whose highest score during the study period was IHC 0 were classified into three groups based on their documented percentage of membrane staining. These subgroups were as follows: IHC 0 absent membrane staining (0% staining); IHC 0 with membrane staining (0% < staining ≤10%, referred to as ‘HER2-ultralow’ or IHC 0+); and percent staining not documented (if

NLP algorithm did not detect any mention of numeric percent staining). We also conducted a sensitivity analysis wherein patients were classified into subgroups based on the lowest IHC score documented during the study period.

Statistical methods

Frequencies and proportions of patients in each HER2 IHC subgroup were calculated for all patients in the analytic cohort. These analyses were repeated among patient subgroups defined by their breast cancer stage—early, locally advanced, and metastatic—at the time of IHC score (within +/− 90 days of IHC score). Early-stage breast cancer was defined as American Joint Committee on Cancer (AJCC) stages IA, IB, IIA, IIB, or IIIA or corresponding TNM staging. Locally advanced breast cancer was defined as AJCC stages IIIB and IIIC or corresponding TNM staging. Metastatic breast cancer was defined as ICD-10 codes C77, C78, C79; AJCC stage IV; TNM M1; or NLP-derived “Stage IV” or “M1” disease. Patient demographic and clinical characteristics were summarized by cross-stratified HER2 IHC, percent staining, and breast cancer stage subgroups using frequencies and proportions or mean (standard deviation) as appropriate.

Online Resource 2

Methods for HCP surveys and interviews

Study participants

The survey and interview analysis consisted of responses from US community-based pathologists and oncologists recruited through GRN and IQVIA's Healthcare Professional (HCP) panel. HCPs who self-reported involvement in biomarker testing and/or treatment of breast cancer patients in a community setting in the US were eligible for inclusion. Pathologists were classified as general or specializing in breast cancer or surgical pathology. Oncologists were either medical oncologists or breast oncologists.

HCP surveys

Two separate structured web-based surveys were developed (one for oncologists, one for pathologists) using Qualtrics software with input from subject matter experts in pathology, oncology, epidemiology, and qualitative methodology, supported by preliminary results from the retrospective cohort EMR analysis. The surveys were pilot tested before being deployed to pathologists and oncologists in US community-based practices within GRN and IQVIA's HCP panel.

The surveys included questions on practitioner characteristics, testing practices, and treatment preferences. Survey responses were collected between February 2024 and May 2024. Survey respondents were then given the option to participate in semi-structured interviews to gain deeper insights and context into their testing and treatment practices and preferences.

HCP interviews

Interviews were conducted via Microsoft Teams from March 2024 to May 2024, each lasting 30–45 minutes. Interview guides tailored to pathologists or oncologists were developed with input from subject matter experts and annotated with HCPs' survey responses prior to the interview to facilitate discussion and minimize duplication in questioning. The interview questions were also informed by preliminary findings from the retrospective EMR analysis to probe deeper into the gaps in HER2 reporting practices. The interview guides were used to ensure consistency in coverage of relevant topics such as practitioner characteristics, HER2 testing orders and clinical decision-making, and emerging technologies.

Data analysis

Survey responses were analyzed within Qualtrics software and Microsoft Office Excel to produce descriptive summary statistics of the data. The interviews were analyzed using MAXQDA software, where a thematic analysis approach was utilized to highlight key themes and patterns in the data. Coding was performed independently by three researchers to ensure reliability; discrepancies were discussed and reconciled across all three researchers.

Online Resource 3

ISH result by breast cancer stage among patients in HER2-negative cohort with IHC 2+ score based on highest IHC score documented during the study period

**Among patients at all HCOs after exclusion criteria are applied**				
**IHC score**	**All HER2-negative patients n=13,100**	**Early-stage HER2-negative patients [a] n=10,746**	**Locally advanced HER2-negative patients [a] n=355**	**Metastatic HER2-negative patients [a] n=1,999**
**IHC 2+**	2,289 (17.5%)	1,786 (16.6%)	73 (20.6%)	430 (21.5%)
**ISH result [b]**				
Negative	1,801 (78.7%)	1,444 (80.9%)	51 (69.9%)	306 (71.2%)
Equivocal, negative	16 (0.7%)	11 (0.6%)	2 (2.7%)	3 (0.7%)
Negative, positive	14 (0.6%)	11 (0.6%)	0 (0.0%)	3 (0.7%)
Positive	29 (1.3%)	23 (1.3%)	0 (0.0%)	6 (1.4%)
Equivocal	1 (0.0%)	1 (0.1%)	0 (0.0%)	0 (0.0%)
No ISH result documented	428 (18.7%)	296 (16.6%)	20 (27.4%)	112 (26.0%)

AJCC American Joint Committee on Cancer, *HCO* healthcare organization *HER2* human epidermal growth factor receptor 2, *ICD-10* International Statistical Classification of Diseases and Related Health Problems, 10th Revision, *IHC* immunohistochemistry, *ISH* in situ hybridization

[a] Breast cancer stage within 90 days of IHC test result. Early-stage breast cancer: AJCC stages IA, IB, IIA, IIB, or IIIA or corresponding TNM staging; locally advanced breast cancer: stages IIIB, IIIC, or corresponding TNM staging; metastatic breast cancer: ICD-10 codes C77, C78, C79; AJCC stage IV; TNM M1; or NLP-derived "Stage IV" or "M1" disease

[b] ISH test result within +/− 90 days of IHC test result

Online Resource 4

HER2 IHC scores and ISH results by breast cancer stage in HER2-negative cohort based on lowest IHC score documented during the study period [a]

Among patients at all HCOs after exclusion criteria are applied				
	**All HER2-negative n=13,100**	**Early-stage HER2-negative n=10,758**	**Locally advanced HER2-negative n=350**	**Metastatic HER2-negative n=1,992**
**IHC 0**	4,969 (37.9%)	3,955 (36.8%)	158 (45.1%)	856 (43.0%)
**IHC 1+**	4,562 (34.8%)	3,816 (35.5%)	93 (26.6%)	653 (32.8%)
**IHC 2+ [b]**	1,598 (12.2%)	1,309 (12.2%)	37 (10.6%)	252 (12.7%)
**ISH result**				
Negative	1,403 (87.8%)	1,157 (88.4%)	30 (81.1%)	216 (85.7%)
Equivocal, negative	12 (0.8%)	8 (0.6%)	1 (2.7%)	3 (1.2%)
Negative, positive	10 (0.6%)	9 (0.7%)	0 (0.0%)	1 (0.4%)
Positive	10 (0.6%)	9 (0.7%)	0 (0.0%)	1 (0.4%)
Equivocal	1 (0.1%)	1 (0.1%)	0 (0.0%)	0 (0.0%)
No ISH result documented	162 (10.1%)	125 (9.5%)	6 (16.2%)	31 (12.3%)
**IHC 3+ [c]**	45 (0.3%)	37 (0.3%)	0 (0.0%)	8 (0.4%)
**No IHC score documented [d]**	1,926 (14.7%)	1,641 (15.3%)	62 (17.7%)	223 (11.2%)
**Among patients at HCO #1**				
	**All HER2-negative n=1,079**	**Early-stage HER2-negative n=955**	**Locally advanced HER2-negative n=48**	**Metastatic HER2-negative n=76**
**All IHC 0**	607 (56.3%)	530 (55.5%)	34 (70.8%)	43 (56.6%)
IHC 0 absent membrane staining (HER2-null; 0% staining)	58 (9.6%)	48 (9.1%)	4 (11.8%)	6 (14.0%)
IHC 0 with membrane staining (HER2-ultralow; 0% < staining ≤10%)	172 (28.3%)	156 (29.4%)	4 (11.8%)	12 (27.9%)
> 10%	1 (0.2%)	1 (0.2%)	0 (0.0%)	0 (0.0%)
Percent staining not documented	376 (61.9%)	325 (61.3%)	26 (76.5%)	25 (58.1%)
**IHC 1+**	337 (31.2%)	300 (31.4%)	12 (25.0%)	25 (32.9%)
**IHC 2+ [b]**	131 (12.1%)	123 (12.9%)	1 (2.1%)	7 (9.2%)
**ISH result**				
Negative	131 (100.0%)	123 (100.0%)	1 (100.0%)	7 (100.0%)
**IHC 3+ [c]**	131 (100.0%)	123 (100.0%)	1 (100.0%)	7 (100.0%)
**No IHC score documented [d]**	4 (0.4%)	2 (0.2%)	1 (2.1%)	1 (1.3%)

AJCC American Joint Committee on Cancer, *HCO* healthcare organization, *HER2* human epidermal growth factor receptor 2, *ICD-10* International Statistical Classification of Diseases and Related Health Problems, 10th Revision, *IHC* immunohistochemistry

[a] Breast cancer stage within +/− 90 days of IHC test result. Early-stage breast cancer: AJCC stages IA, IB, IIA, IIB, or IIIA or corresponding TNM staging; locally advanced breast cancer: stages IIIB, IIIC, or corresponding TNM staging; metastatic breast cancer: ICD-10 codes C77, C78, C79; AJCC stage IV; TNM M1; or NLP-derived "Stage IV" or "M1" disease

[b] All patients with IHC 2+ and ISH-positive as lowest score were confirmed to have documentation of HER2-negative status during the study period

[c] All patients with IHC 3+ documented as lowest score were confirmed to have documentation of HER2-negative status during the study period

[d] All patients without a documented IHC score had qualitative documentation of HER2-negative status in their medical record (e.g., HER2 status = 'Negative')

Online Resource 5

Geographic distribution of surveyed HCPs across the US by HCP type [a]

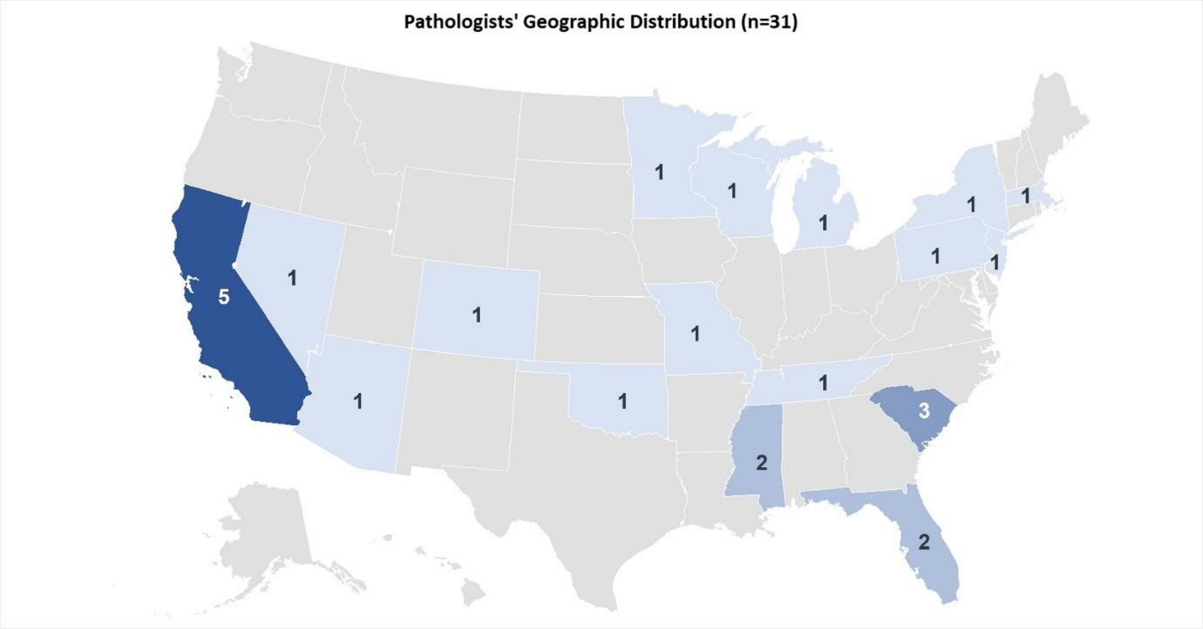

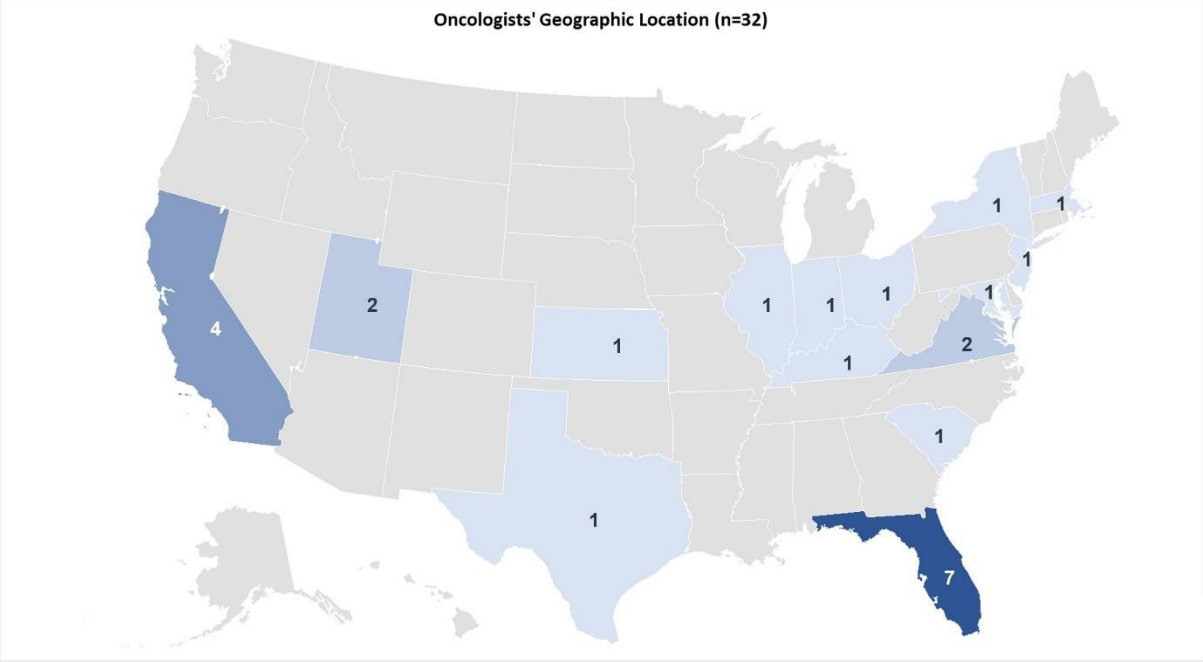

HCP healthcare professional

[a] Not pictured on the maps: of the surveyed pathologists, three were multistate, two were nationwide, and one was unknown. Of the surveyed oncologists, one was multistate and five were unknown
